# Formaldehyde containment efficiency with a next-generation grossing station promising safer use in anatomical pathology practice

**DOI:** 10.2478/aiht-2025-76-3971

**Published:** 2025-06-30

**Authors:** Stefano Dugheri, Giovanni Cappelli, Ilaria Rapi, Niccolò Fanfani, Donato Squillaci, Antonio Baldassarre, Veronica Traversini, Simone De Sio, Nicola Mucci

**Affiliations:** Link Campus University, Department of Life Science, Health, and Health Professions, Rome, Italy; University of Florence, Department of Experimental and Clinical Medicine, Florence, Italy; Careggi University Hospital, Division of Occupational Medicine, Florence, Italy; La Sapienza University of Rome, Rome, Italy

**Keywords:** containment capacity, gas chromatography, occupational safety, optical gas imaging, potassium iodide test, short-term exposure limit, tracer-gas leak test, kapacitet zadržavanja, plinska kromatografija, sigurnost na radu, optičko snimanje plinova, test kalijeva jodida, granica kratkoročne izloženosti, test curenja plina

## Abstract

Healthcare needs to re-evaluate its resources and make the processes more efficient. The pathologist's workspace is often narrow and limits access to grossing information mid-procedure. An ergonomic, open-front containment console – called grossing station – can improve this situation. Besides collecting airborne formaldehyde and chemical fumes, its cupboard with adjustable vertical protective screen simplifies the workflow with a customisable open work surface that allows image acquisition and includes voice recognition and waste dispensers. However, its containment efficiency and compliance with international safety standards has not yet been investigated. The aim of our study was to address this lack of information and propose a standard procedure for testing containment efficacy of next-generation grossing stations. For this purpose we ran the potassium iodide test and a formaldehyde leak test with a new tracer-gas method on a new DFB900 grossing station model and established that its protection factor of 10^5^ complied with the EU standards and the NIOSH safety limits. Future research should include a range of new grossing stations and a wide spectrum of harmful compounds that pose occupational health risk to their operators.

## Abstract (hr)

Redovita procjena resursa i povećanje učinkovitosti postupaka nužnost su u zdravstvenoj skrbi. Radni prostor patologa često je uzak i ograničava pristup informacijama tijekom analize uzoraka materijala. Ergonomska konzola s otvorenim prednjim dijelom – koja se često naziva radna stanica i/ili digestor – može poboljšati ovu situaciju. Osim što sprječava širenje formaldehidnih i kemijskih isparina, njezin ormar s podesivim vertikalnim zaštitnim zaslonom pojednostavljuje rad s prilagodljivom otvorenom radnom površinom koja omogućava snimanje, uključuje prepoznavanje glasa i uređaje za odlaganje otpada. Međutim, njezina učinkovitost u kontroli izloženosti formaldehidu iz zraka i usklađenost s međunarodnim sigurnosnim standardima još uvijek nisu istražene. Cilj našeg istraživanja bio je utvrditi upravo to: koliko su takve stanice/digestori uspješni u zaštiti radnog prostora te predložiti standardizirani postupak za njihovo testiranje učinkovitosti kontrole formaldehida u zraku. U tu svrhu proveli smo test kalijeva jodida (pomoću KI disk metode) i test ispitivanja koncentracije formaldehida u zraku pomoću nove metode praćenja curenja plinova na modelu radne patološke stanice/digestora DFB900 te ustanovili da je njezin zaštitni faktor 10^5^ i da udovoljava EU-ovim standardima i zaštitnim ograničenjima NIOSH-a. Buduća istraživanja trebaju obuhvatiti niz novih radnih stanica za anatomsku patologiju i širok spektar štetnih spojeva koji predstavljaju rizik za zdravlje radnika u patološkim laboratorijima.

The potential carcinogenicity of formaldehyde (1) has prompted new strategies aimed at improving grossing operations at anatomical pathology laboratories. Since 12 July 2024, the healthcare sector has to comply with the new and stricter occupational exposure limit (OEL) for formaldehyde exposure of 370 mg/m^3^ (2).

Pathologists spend long hours grossing (3), which means that they inspect specimens to retrieve diagnostic information. Pathologists usually receive surgical specimens in a container filled with formalin, an aqueous solution of formaldehyde acting as fixative, whose concentrations range between 4 % and 10 % (4). The first step is to orient and describe the specimen in detail by measuring, weighing, and photographing it. Next, the tissue is carefully dissected and sampled. Tissue samples are then embedded in paraffin and placed in cassettes to obtain blocks to be cut further into slides for microscopy. Every step of the procedure is documented along the way.

Until recently, the only marketed grossing stations were benches with aspiration hoods or cupboards with adjustable vertical protective screen (5). While these provide protection from the fumes, they poorly adjust to the ergonomic needs of operators and are not modular enough to accommodate upgrades with new technologies, such as a dictaphone or a digital recording system. Because of these limitations, lab assistants are needed to record dictated findings, which raises the cost of operation and, more importantly, exposes more people to formaldehyde. Furthermore, several studies (6,7,8,9) have found that the error rates in pathology labs range from 1–43 %, which are sometimes owed to transcription (10) or post-coding errors (11), often producing wrong clinical history, and incomplete or incomprehensible diagrams. Recent statistic from Italy shows that each medical error across department costs the healthcare system 96,831 euros in average, with an annual rise of 4 % (12).

The latest generation of grossing stations, however, brings vast improvements, which drive their sales growth, projected to rise 7.2 % annually from 2024 to 2033 (13). New grossing stations provide a multi-functional, ergonomic, safe, and practical work area. Skin exposure to splashes is minimised thanks to the mobile splash screen, and installed open-fronted containment systems (relying on laminar and/or back down-draft ventilation) protect from fume exposure (14). Recent years have also seen the development of a high-performance, cost-effective digital optical console, and introduction of voice recognition technology to replace dictation to assistants (15, 16). These new, flexible, and efficient consoles incorporate modular architecture, connectivity, appropriate software, and a digital information system that records whole images of specimens (15, 17).

However, safety and performance of the next generation of grossing stations have not yet been evaluated and tested, and manufacturers often omit these evaluations.

The aim of this pilot study was therefore to address this gap by testing the microbiological safety and efficiency in containing formaldehyde fumes of one such new open-fronted containment grossing station and to propose a new standard procedure for such testing.

## MATERIALS AND METHODS

### Grossing station

**Figure 1:**
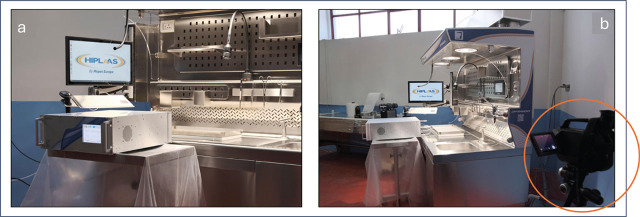
Test to assess formaldehyde gas containment on the worktop of the DFB900 grossing station: a) pre-calibrated laser infrared spectrometer; b) optical gas detection thermal camera

For the microbiological safety and formaldehyde containment performance test we selected the DFB900 grossing station manufactured by HIPLAAS (Montefusco, Avellino, Italy) (Figure 1), with a 150×90 cm stainless steel worktop, adjustable in height from 80 to 110 cm. It has a dual-draft ventilation system configured to combine down-draft (airflow is directed downward) and back-draft (airflow is directed from the user to the back of the station) with a total extraction rate of 1200 m^3^/h. In addition, a gentle frontal air curtain flows down from the top of the station in front of the operator. This grossing station is equipped with video recording and imaging systems, integrated LED lights, mobile splash-shield in transparent Lexan resin, and wheels with parking feet.

### Potassium iodide test

To test the grossing station for microbiological safety we used the KI-Discus^™^ potassium iodide test (CTS Europe Ltd., Portsmouth, UK) (18) as described by Nicholson et al. (19) to see if it meets minimum performance requirements set by the European Standard EN 12469:2000 for safety cabinets for work with micro-organisms (20). Briefly, a fine mist of potassium iodide droplets produced by a spinning disk is used as a challenge aerosol to measure containment. Five centripetal collectors sample in four runs the air and deposit any present potassium iodide particles on filter membranes. At the end of sampling, the filter membranes are placed into a solution of palladium chloride. If present, potassium iodide forms clearly visible grey/brown particles, which are then counted, and the aperture protection factor (A_pf_) of the safety cabinet calculated as follows:

[1] $$\mathrm{Apf}=\frac{62\times 10^{5}}{\text{n}}$$

where *n* is the number of potassium iodide particles recovered in the filter. The A_pf_ is the ratio of exposure to airborne contamination on the open bench to exposure within the containment cabinet under test. The safety A_pf_ threshold should not be lower than 10^5^, i.e., the device has failed the safety test if more than 62 particles are counted on the filter paper (21).

### Tracer-gas leak test for formaldehyde

To evaluate the grossing station's formaldehyde containment capacity against leaks we designed a new tracer-gas method. Briefly, we placed a 60×40×5 cm stainless steel tank containing 300 mL of formalin (38 % formaldehyde solution in water) on the work surface and kept stirring for 4 h. Airborne formaldehyde was measured with a ProCeas^®^ formaldehyde analyser (AP2E, Aix-en-Provence, France), which is a pre-calibrated laser infrared spectrometer with a response time of 2 s and detection limit of 0.12 μg/m^3^ (Figure 1a). The concentration of airborne formaldehyde was measured both with and without the ventilation system activated after 30 min of equilibrium at 20 °C at 10 cm from the emission source on the worktop as well as at 15 frontal and lateral points positioned 10 cm outside the grossing station worktop.

Furthermore, to visualise the real-time evacuation path of airborne formaldehyde on the worktop we used a FLIR GFx320 optical gas imaging (OGI) camera (FLIR Systems, Nashua, NH, USA) with high sensitivity and precision in the temperature range between −20 °C and +350 °C (Figure 1b).

Besides direct measurements mentioned above, we ran indirect formaldehyde measurements in front of the grossing station by collecting six 15-minute air samples with a GasCheck Pro automatic collector box (AMS Analitica, Pesaro, Italy) equipped with a GSM module (set to 1.2 L/min flow rate) and FFA–Sep-Pak XpoSure 2,4-dinitrophenylhydrazine (DNPH) cartridges (Cat. No. WAT047205, Waters, Milford, MA, USA). The collected air samples were then analysed for formaldehyde using our method described elsewhere (22). Briefly, formaldehyde-2,4-DNPH was injected into a 35 % phenyl 65 % polydimethylsiloxane (PDMS) stationary phase column (Cat. No. 122-3832UI, Agilent, Santa Clara, CA, USA) of the Varian CP-3800 gas chromatograph with a thermionic specific detector (TSD) (Varian, Walnut Creek, CA, USA).

## RESULTS AND DISCUSSION

**Figure 2:**
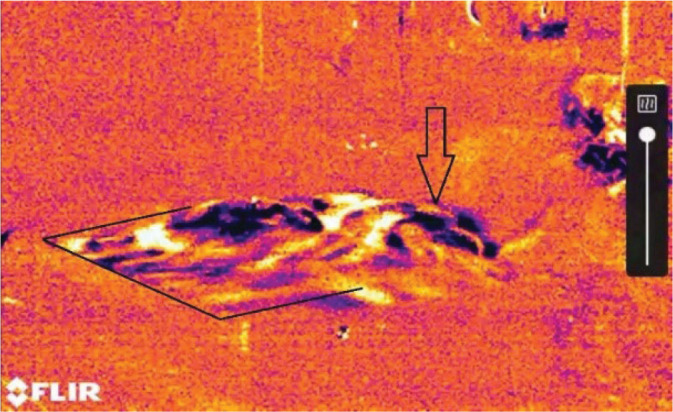
Movement of formalin vapour (black arrows) by the grossing station ventilation system recorded with the optical gas imaging camera

The new DFB900 grossing station has many features that improve the quality of work, but there is no standard to evaluate its containment capacity for formaldehyde. The available EN14175-3:2019 standard for the definition of containment of conventional chemical fume hoods (23) is not fit for this grossing station as it does not have the vertical screen sash. This is why we found the KI-Discus test the only able to define containment for this kind of grossing station. The station passed the potassium iodide test (Table 1), as the protection factor was always higher than the safety threshold of 10^5^ set by the EN 12469:2000 standard (20). As for the tracer-gas measurement, formaldehyde concentration at 10 cm from the source (the stainless steel tank with 300 mL of formalin on the gross station worktop) was 27.6±3.6 mg/m^3^ with ventilation off, which is above the 24 mg/m^3^ threshold for categorisation as immediately dangerous to life and health (IDLH) by the National Institute for Occupational Safety and Health (NIOSH) (24). However, with ventilation on this level was consistently lower than 10 μg/L at all 15 measuring points, indicating a massive decrease in formaldehyde concentration. The OGI camera also showed that all the vapours produced on the worktop were immediately aspirated by the back- and down-drafts (Figure 2). Gas chromatography showed that formaldehyde concentrations, ranging between 4.9 and 11.1 μg/m^3^ (median 8.6 μg/m^3^), in the six collected 15-minute samples were compliant with the NIOSH 15-minute short-term exposure limit (STEL) of 123 μg/m^3^ (24).

**Table 1:** Potassium iodide (KI-Discus) test results for the HIPLASS DFB900 grossing station

**Test**	**A_pf_**	**Results**	**Note**
1	>10^5^	1.19×10^5^	Passed
2		1.15×10^5^	Passed
3		1.15×10^5^	Passed
4		1.15×10^5^	Passed
5		1.17×10^5^	Passed

A_pf_ – aperture protection factor

### Study limitations

A limitation of our study is that it only analysed the containment of formaldehyde, as this new grossing station has specifically been designed for work with anatomical specimens stored in formalin solutions. However, these workstations could have a variety of applications, so a future study investigating the containment capabilities of other hazardous compounds would be highly valuable.

## CONCLUSION

Despite its limitations, our pilot study combining the potassium iodide, trace-gas, gas chromatography, and optical tests has confirmed that the new generation of grossing stations, represented by the HIPLASS DFB900 model, meets the main safety standards for formaldehyde exposure and, also thanks to its innovative technologies improving the work process and ergonomics, brings a promising advancement for pathological anatomy or forensic medicine laboratories. Future research should include a range of new grossing stations and a wide spectrum of harmful compounds that pose a biological hazard to their operators.
